# New Strategies for Endometrial Cancer Detection and Management

**DOI:** 10.3390/ijms24076462

**Published:** 2023-03-30

**Authors:** Laura Paleari

**Affiliations:** Research, Innovation and HTA Unit, A.Li.Sa., Liguria Health Authority, 16121 Genoa, Italy; laura.paleari@alisa.liguria.it; Tel.: +39-010-5484243

With 400,000 new cases and over 80,000 deaths a year worldwide, endometrial cancer (EC) holds a rather unfortunate record, namely, that of the tumour with the highest increase in incidence, a unique trend among gynaecological cancers [1]. EC is more common in high-income countries where obesity rates are high, and this cancer is the fourth most common malignancy in women. It mainly affects postmenopausal women, where patients’ mean age at diagnosis is 61 years, and most cases are diagnosed in women aged 45 to 74 years [1]. The prognosis is worse in older patients with higher-grade and more prevalent tumours, with a median 5-year survival rates of 95% for stage I/II and of 60% for stage III/IV. Overall, 63% of patients are tumour free ≥5 years after therapy [1].

To date, the standard management of early-stage cancer includes surgery, radiotherapy and/or chemotherapy. Thus, there is a clear unmet medical need for disease management, and it is essential to find a molecular target to better classify ECs in terms of prognosis and to drive the therapeutical choice. The intention of this Special Issue is to increase our understanding of EC development, which may lead to the discovery of new molecular diagnostic technologies and targeted therapeutics. For instance, although EC is a hormone-dependent neoplasm, there are no recommendations for the determination of steroid hormone receptors [2]. Currently, only a few studies have assessed the impact hormonal therapy (HT) has in EC treatment, suggesting how HT is a useful treatment modality to prolong progression-free survival (PFS) and overall survival (OS) [3]. Recently, several studies have assessed diagnostic and therapeutic markers. Bioinformatics analyses indicated that NRAS gene deregulation affected the OS rate of patients with EC, leading to prognostic significance [4]. Celsi and colleagues with a gel-based proteomic assay showed that suprabasin (SBSN), an oncogene previously associated with poor prognosis in different cancers, could be a potential novel biomarker in EC [5]. Interestingly, the study of Benbrook et al. analysed serum from EC patients (with or without lymph node metastases, and compared with that of benign gynaecological surgery patients) to determine if it is possible to predict the presence of positive lymph nodes, which represents a critical factor guiding treatment decisions [6]. The results suggest that pathways implicated in metastases included the loss of PTEN, PI3K, AKT and PKA activation, leading to calcium signalling, oxidative phosphorylation and oestrogen receptor-induced transcription, platelet activation, the epithelial-to-mesenchymal transition, and senescence. Upstream activators implicated in these events included neurostimulation and inflammation, the activation of G-protein-coupled receptor Gβγ, the loss of HER-2 activation and the upregulation of the insulin receptor [6].

Moreover, advancements have newly been made in EC pathological classification through the new molecular classification that allows for a more accurate categorisation of the pathology, which is useful for making a therapeutic choice. In fact, for decades, EC risk stratification has been based on histopathological features, such as tumour grade and histotype, depth of myometrial invasion, and cervical and adnexal involvement. The Cancer Genome Atlas (TGCA) has proposed a new and disruptive molecular classification of ECs which identifies, based on molecular characteristics and expression, four distinct subgroups. This approach has revolutionized diagnostics, allowing data to be refined from a precision medicine point of view through the definition of a real therapeutic algorithm [7,8].

Furthermore, in recent years, vibrational biospectrometry methods for the screening and diagnosis of EC have gained ground. Vibrational biospectroscopy is a noninvasive objective technique that uses the interaction of light with tissue to obtain detailed information about the chemical composition of biological samples. Current diagnostic strategies are time-consuming, invasive and have limited accuracy, and there is no population-level screening. Treatment depends on the patients’ health, type of disease and prevalence at the time of diagnosis. Most women will be offered surgery; however, the role of lymph node dissection in the early stage of the disease remains controversial. There is, therefore, a need for a test that is objective, accurate, and capable of detecting pre-cancer and early cancer, as well as able to identify metastatic lymph node involvement, so that lymph node excision is performed only when necessary. However, while there has been significant progress made in vibrational spectroscopy development, the techniques are still at the trial phase and have not yet been translated into clinical practice [9].

Moreover, for over 20 years, the availability of a new therapy capable of affecting the prognosis of a tumour which, although not considered particularly aggressive, had a proportion of recurrences that were difficult to treat, had not been verified. In the last year, interesting advances have taken place with the introduction of immunotherapy for the treatment of ECs. The drug dostarlimab is an antibody that blocks the PD-1 receptor, and is indicated for adult patients with advanced or recurrent EC with a mismatch repair/microsatellite instability system defect. This immunotherapy drug, in fact, recently had accelerated approval by the US FDA, and the European Commission itself gave the green light to its conditional marketing. This approval is based on results obtained through the GARNET multi-cohort study [10,11]. The data collected showed that the dostarlimab treatment resulted in an objective response rate (ORR) of 43.5% in 71 patients with a DNA mismatch repair deficiency, including a complete response rate (CRR) of 12.7% and a partial control rate (PCR) of 29.6%. A duration of the response at six months was seen in more than 93.3% of the respondents [10,11].

The main aim of this Special Issue is to provide an open-source sharing platform for significant works in the field of molecular oncology that can increase our understanding of EC development, and which may lead to the discovery of new molecular diagnostic technologies and targeted therapeutics.
